# Antibiotics and Allergic Disorders in Childhood

**DOI:** 10.2174/1874434600802010048

**Published:** 2008-05-22

**Authors:** Sue Jordan, Mel Storey, Gareth Morgan

**Affiliations:** 1 School of Health Science, Swansea University, Swansea, SA2 8PP, UK; 2 School of Medicine, Swansea University, Swansea, SA2 8PP, UK

**Keywords:** Allergic disorders, antibiotics, prescribing, adverse drug reactions, evidence based practice.

## Abstract

**Aim:**

This paper explores the possible association between antibiotics prescribed in infancy and allergic disorders, mainly eczema and asthma, in childhood.

**Background:**

No-one fully understands why childhood asthma and eczema have become so common. Some authorities suggest that there may be an association between eczema and asthma and antibiotics prescribed in childhood; however, others disagree.

**Method/Evaluation:**

The available literature was reviewed to examine the links between prescribed antibiotics and childhood eczema and asthma.

**Findings/Key Issue:**

Some, but not all, research indicates that antibiotic administration in pregnancy, childbirth or infancy may be linked to childhood asthma and eczema, but much uncertainty remains. None of the papers identified stated the doses of antibiotics prescribed. In addition, we were unable to locate studies reporting the interactions between antibiotics and the developing immune system.

**Conclusion:**

Health care professionals should be selective when prescribing antibiotics. Further prospective work is needed to guide the prescribing of antibiotics in childbirth and infancy.

Health care professionals, particularly nurses, are encouraged to base their practice decisions on research evidence. However, some commentators suggest that this has limitations [1,2]. One limitation of the ‘evidence-based paradigm’ is the absence or paucity, and, consequently, possible bias, of evidence to guide decision-making in some circumstances.

Ideally, every practice activity would be based on the results of randomised, multiple blind, parallel group, placebo controlled pragmatic clinical trials of adequate size, supported by large cohort studies and service users’ views. While these standards are sometimes achieved by those investigating the benefits of drugs, there has been no comparable investment in research into the adverse side effects of drugs. As an example, this paper discusses a putative adverse drug reaction of antibiotics: asthma and eczema in childhood.

## ALLERGIC DISORDERS IN CHILDHOOD

Childhood allergic disorders (mainly asthma, eczema and hayfever) are becoming increasingly common and increasingly severe [3,4]. Each year, 12 million people in the UK receive treatment for allergic disorders [4]. The prevalence of allergic disorders in childhood increased in the latter half of the twentieth century: by 1995, 53.9% of UK 12-14 year olds (n=27,507) had experienced asthma, hayfever or eczema [5], and 41% of 10 year olds had experienced eczema (n=1456) [6].

Children with even mild wheezing and asthma lose sleep due to distressing nocturnal symptoms and 1.5% UK children are kept awake at least 1 night each week by wheezing [4]. Asthma accounts for 128,604 disability adjusted life years in European children aged 0-4, more than any other single condition and more than all malignancies in that age group [7]. The most severely affected children are frequently admitted to hospital, in acute distress, struggling to breathe. If urgent preventative action is not taken, asthma deaths worldwide will increase by 20% in the next 10 years [8].

Overall, eczema may impose a greater burden on families than type 1 diabetes [9]. Skin conditions account for 9,033 disability adjusted life years in European children aged 0-4 [7]. Children affected by eczema are unable to sleep or concentrate, due to itching: 3% of UK children are kept awake at least 1 night each week by eczema [4].

The lives of most sufferers are further restricted by their medications. Most children with asthma or eczema receive corticosteroids or immunosuppressants, which can have adverse effects, including increased vulnerability to infections, and further disturbance of body image. The need for rigorous compliance with therapeutic regimens also curtails the child’s lifestyle.

The burden of atopic disease is falling disproportionately on children in developed countries, where the prevalence of infectious illness is relatively low [10].

Epidemiological trends, taken with observation studies [11], indicate that modern lifestyles may have disrupted the long-standing commensal relationships between humans and their microflora: the hygiene hypothesis [12,13]. Children in rural Eastern Europe have, to date, largely escaped the ‘allergy epidemic’ [14]. However, the culpable environmental components of the modern hygienic environment have not been identified. It has been noticed that the increase in allergic disorders co-incided with the introduction of antibiotics. In the developed world, the prevalence of allergic disorders in childhood may no longer be rising [15, 16], whilst, simultaneously, the prescribing of antibiotics in primary care is declining [17, 18]. When juxtaposed with biologically plausible mechanisms, these contemporaneous trends circumstantially support the association between antibiotics and childhood allergy.

## BIOLOGICAL MECHANISMS

Causal associations between events and disease may be considered with reference to the Bradford Hill criteria [19]: strength, consistency, specificity, timeframe and coherence of association, the dose-response relationship, biological gradient and plausibility, experimental evidence, analogies and precedents. There may be a biological basis for the association between antibiotics in childbirth or infancy and allergic disorders in childhood.

Allergic disorders are associated with atopy [glossary], which occurs when the regulation of the immune system is upset and lymphocytes produce IgE antibodies, rather than IgG antibodies [glossary]. IgE antibodies trigger release of histamine and other inflammatory mediators, which give rise to the signs and symptoms of inflammation. The regulation of the immune system depends on the balance between the 2 main types of T helper cells [glossary]: Th1 and Th2. If the balance deviates towards the Th2 cells, away from those of the Th1 cells, atopy develops. At birth, the Th2 cells predominate. Normally, environmental factors shift the balance of the immune system away from Th2 cells towards Th1 cells, which prevent the development of allergies. However, this change does not occur in children with atopy [20]. This is supported by the observation that children experiencing fever in early life, which stimulates the Th1 cells, are less vulnerable to allergy [21].

In today’s hygienic environment, children, and their immune systems, may be under-exposed to certain microorganisms, particularly those that normally inhabit the gut, which have been closely associated with the development of the immune system of the gastro-intestinal tract during evolutionary history. Previous generations of children encountered these microbiological ‘old friends’ routinely [22]. At birth, the neonate’s gastrointestinal tract is rapidly colonised by micro-organisms, mainly from the mother. This colonisation stimulates the lymphoid tissue lining the gut, which contains at least 60% of the body’s lymphocytes, and drives the development of the neonate’s immune system [23, 24]. However, the bacteria colonizing the neonatal colon have changed since the 1970s and 1980s, with a decrease in gram negative organisms, such as *E. coli*, and an increase in gram positive microflora, such as staphylococci. Since the 2 groups of micro-organisms affect the gut’s immune system in different ways, this may have implications for the development of allergic disorders [25].

Caesarean delivery [26], antibiotics administered to women antenatally [27, 28], or in the peripartum period [29] or to infants [30-32] may change the initial colonisation and the development of the neonatal gut microflora. This could affect the regulation of the immune system, predisposing the child to develop allergic disorders [33]. Consequently, an association between antibiotics, stool flora, the neonatal immune system and the subsequent development of allergy has been postulated [34]. However, the impact of prescribed medications on the Th1/Th2 cytokine [glossary] balance regulating allergy and atopy has not been explored [35, 36].

## AIM

This paper explores the possible association between antibiotics in early life and allergic disorders in childhood.

## METHODS AND SEARCHES

To identify the available evidence, Medline was searched during February 2008, using search terms: *antibiotics *AND *childbirth/ labour/ parturition* AND *allergy/ atopy/ eczema/ asthma*. Two articles were retrieved, one of which was relevant. The search terms: *antibiotics *AND *eczema/ asthma/ atopy* AND *adverse drug reactions/ adverse effects,* restricted to ‘all children’ and English language yielded 34, 95 and 31 articles, respectively, of which 9, 17 and 8 were relevant data papers, and 23, 64 and 19 were on unrelated topics. Repeating the searches on Embase identified a further 2 relevant articles. When the Cochrane database was searched with the terms *antibiotics* AND *allergy/ allergic disorders/ eczema/ asthma,* no relevant reviews were identified. Reference lists were searched for further studies. Most articles retrieved by these terms related to acute responses to antibiotic administration, indicating the relatively low profile of the adverse drug reaction under consideration.

Studies and methods employed were described (Tables **1** and **2**). All studies were observational: no experimental work was identified. There is no consensus as to appropriate quality indicators for adverse event research, as discussed below; therefore, we included all papers containing relevant data. The diversity of populations studied and methods employed precluded any statistical analyses.

## EXISTING STUDIES

The largest available dataset indicated that antibiotic administration in the first year of life increased the risk of GP diagnosis of eczema before age 11. However, there were inconsistencies in the dose-response relationship: one course of antibiotics increased the risk of eczema (adjusted hazard ratio (aHR) 1.22 (95% CI 1.12-1.34), but four or more did not [37]. Also, this study did not account for antibiotic doses, home environment, family history and laboratory parameters. Farooqi and Hopkin (1998) reported a similar association with antibiotic use in the first 2 years of life [38]. Most retrospective studies have indicated an increased risk of childhood eczema if antibiotics are administered within the first 1-3 years of life [34, 38-43]. Others have found an association with antibiotics in pregnancy, but not in the first year of life [44], while another medical record study [45], a case-control study with no figures [46] and a prospective cohort [47] found no association (Table **1**).

In the main, these are retrospective studies, relying on questionnaires administered to parents, asking for recall of administration of antibiotics up to 6 to 10 years previously. Information collected from participants is vulnerable to recall bias and, if collected more than 2 weeks after the event, forgetfulness [48], which concurs with our experience that many parents are unable to recall accurately the names, doses and usage of antibiotics over a 6 week period. Most retrospective questionnaire data collection in this area has used the ISAAC questionnaire. A retest of this questionnaire by 347 parents within 12 months gave a kappa value of 0.63 (95%CI 0.57-0.69) for reports of antibiotic usage in the first 3 years of life, indicating incomplete agreement between parents’ initial response and the re-test [40].

More work has been done on asthma, and the findings are less equivocal (Table **2**). A meta-analysis of 8 studies indicated that use of any antibiotics in the first year of life was significantly associated with childhood asthma. The association was stronger in retrospective than prospective cohorts (OR 2.82 (2.07-3.85) *versus* OR 1.12 (0.88-1.42)), highlighting the need for further prospective work [49]. Also, the largest study suggested a dose/response relationship: antibiotic administration in the first year of life increased the risk of GP diagnosis of asthma before age 11, depending on the number of courses prescribed: HR 1.26 (1.13-1.40) for one course, and HR 1.99 (1.72-2.31) for four or more courses [37]. Most studies support these findings [34, 38-42, 50, 51]. However, the association is refuted by a body of research, mainly that relying on parental report of antibiotic use [52, 53], but also two record reviews [45, 54], and a case-control study found an association for use in the first 3 years, but not the first year [55]. Although children living on farms in Eastern Europe are relatively immune from allergic disorders, asthma is more common amongst children on farms in the American mid-West where antibiotics are added to animal feed [56].

Some studies which linked antibiotics to asthma or eczema found no association between antibiotics and atopy (assessed by skin prick tests or measurement of IgE antibodies in venous blood) [34, 40, 41, 50, 51] (Table **3**).

Allergic disorders and atopy tend to run in families, but only smaller studies have focused on families at high risk of allergic disorders (n=1460, 448, 937, total 2845) [39, 52, 53], and the largest of these was retrospective. The impact of antibiotics may be limited to certain subgroups, for example, homes with <2 pets, infants breastfed >4months [57], or a family history of allergy (OR for eczema 1.6 95%CI 1.0-2.6) [41]. There are suggestions that type of antibiotic is important, with broad-spectrum antibiotics having a stronger association [37, 38].

## ANTIBIOTICS AND PROBIOTICS

The bacteria which colonise the sterile gastrointestinal tract of newborn infants include lactic acid producing bacteria, enterobacteria, streptococci and staphylococci [58]. Where lactic acid producing bacteria are relatively deficient in the gut microflora, the neonatal immune system develops suboptimally, leaving the infant susceptible to allergic disorders [59].

Probiotic food supplements contain lactic acid producing bacteria and are known to affect the neonatal gut flora. It is suggested that this induces the maturation of Th1 cells, which inhibit the development of the signs and symptoms of allergy [60, 61]. The potential of prophylactic probiotics to prevent allergic disorders [60, 62] is supported by a meta-analysis of 5 clinical trials with atopic dermatitis/ eczema as the primary outcome (relative risk 0.61 (0.49-0.74 in a fixed effects model, n=1406) [63]. Clinical trials of infant formula feeds containing probiotic organisms are underway [64, 65]. However, there are concerns that any benefits probiotics confer could be negated by antibiotics, which disrupt the gut flora [39, 66, 67].

## EVIDENCE FOR ADVERSE EFFECTS

There is no single hierarchy of evidence or a single ‘gold standard’ research method for the identification of the adverse effects of treatment [68]. Even the best-funded randomised controlled trials are rarely powered to detect adverse effects [69, 70], and the best-conducted trials may be confounded or obfuscated by cross-over [71].

To date, ethical and practical considerations, not least the time between antibiotic administration and the onset of allergic disorders, have precluded investigation of this adverse drug reaction by clinical trials. Causal relationships between medication and adverse events cannot always be formally tested by experimental research, and may retain an element of subjective interpretation [68, 72, 73]. Therefore, the dose-response relationship remains pivotal in identifying adverse drug reactions [74] and establishing causality [19, 75]. Although the putative association between antibiotics and allergic disorders has been explored by a variety of non-experimental methods (Tables **1**-**3**), none of these studies have recorded doses prescribed or obtained detailed information on the balance of the immune system and its cytokines, leaving some of the Bradford Hill [19] criteria unfulfilled.

Antibiotic administration is unlikely to be the only cause of allergic disorders. There are several possible explanations for the increased incidence in allergic disorders that coincided with the introduction of antibiotics: genetic predisposition, infant feeding, urban living and other environmental factors are likely to be involved. Associations uncovered in non-experimental observational research do not allow attribution of causation: children vulnerable to allergic disorders may also be in greater need of antibiotics, and children with asthma are prescribed more antibiotics than their contemporaries [76]. Although children wheezing at age 8 were prescribed more antibiotics, the association almost disappeared when only antibiotics for non-respiratory infections were considered (OR 1.05 CI 1.00-1.10) [53]. In contrast, other work indicated that the association remained when antibiotics for lower respiratory tract infections are excluded [38].

Asthma is associated with repeated upper respiratory tract infections in childhood [77]. While some studies indicate that children with atopy suffer more viral infections [38], the largest UK cohort found no association between infections and allergic disorders [37], and low numbers of respiratory tract infections, allergies and antibiotic prescriptions characterise childhood in rural Poland [14]. The effect of antibiotics on allergic disorders is more marked for early exposure [37]. Crucial development of the immune system occurs in very early life [20]. Therefore, even large datasets without peripartum data, such as GP records, may fail to identify associations.

## DISCUSSION

Allergic disorders remain a major public health problem, but their aetiology is largely unknown. Antibiotic prescribing has been extended to nurses and other health care professionals, devolving responsibilities from doctors to healthcare professionals who may have received a less detailed education and preparation in pharmacology and immunology, or may be more vulnerable to pharmaceutical industry promotions [78]. Antibiotics save lives and cure infections, but they can be over-prescribed [79, 80]. When prescribed for sore throat, ear or upper respiratory tract infections, antibiotics reduce the risk of serious complications, but, in UK general practice, over 4,000 patients need to be treated to prevent one complication [81]. However, withholding therapy from severely ill patients may jeopardise the management of potentially serious or life-threatening infections [82, 83]. Delayed administration of antibiotics in patients with confirmed bacterial meningitis increases the risk of adverse outcomes [84]. For every 100 courses of antibiotics prescribed to treat chest infections in children, one case of pneumonia is prevented [81]. There are concerns that therapeutic Calvinism may be responsible for the rising incidence of rheumatic fever and renal damage secondary to streptococcal infections in children aged 4-15. Consequently, a more selective approach to antibiotic therapy for sore throats, using laboratory tests, is advocated [85]. This approach reduces antibiotic prescribing for children 3-17 with sore throats, but without reducing overall paediatric primary care antibiotic prescribing [18].

If antibiotic administration is to be rationalised, professionals and patients need full information of their risks and benefits or harms. Most antibiotic prescribing takes place in primary care, where there is considerable variation between practitioners [86]. Interventions to reduce antibiotic prescribing have been successful [87], which might indicate that scope for practice modification exists. Current guidelines and quality indicators for antibiotic prescribing focus on bacterial resistance, and offer no consensus [88, 89], reflecting tensions between the needs of individual patients and public health priorities [90]. However, due to the paucity of evidence, current guidelines take little account of allergic disorders. Those developing prescribing guidelines should not regard absence of evidence as equivalent to evidence of absence when considering the risk of adverse drug reactions.

Allergic disorders in childhood disproportionately afflict children from disadvantaged backgrounds [87], particularly the urban poor of the USA. The high incidence of allergic disorders amongst children of large families, living in overcrowded and rat or cockroach-infested homes reduces the plausibility of the ‘hygiene hypothesis’ [91]. Poorer pre-school children are more likely to be prescribed antibiotics in primary care for viral infections [92], and at an earlier age [93]. Therefore, children from disadvantaged backgrounds will benefit most from modifications in GP prescribing.

The public health importance of this putative association indicates that this issue requires further exploration. It is important to capture and disseminate the impact of prescribing while the data is relatively contemporary. Previous studies are based on the prescribing patterns of the 1980s [38, 40, 41, 50, 56] or earlier [49]. Changes in GP prescribing of antibiotics 1995-2000 changes in GP prescribing of antibiotics 1995-2000 [1] and epidemiological trends [15, 16] indicate that previous work may be obsolescent.

## IMPLICATIONS

When caring for children who have received numerous courses of antibiotics and whose mothers received antibiotics in pregnancy, practitioners should be alert for the signs and symptoms of eczema and asthma and consider prompt referral.

Professionals, including nurses, making prescribing decisions, or planning follow-up patient monitoring and surveillance [94], need evidence on both the benefits and harms of medications. Evidence-based medicine is only valuable to the extent that the evidence base is complete [95]. Without prospective studies, the temporal link between respiratory tract infections, administration of antibiotics and allergic disorders and the appearance in the defect in Th1 maturation associated with allergic disorders [96] will remain unknown. If research funding, and research effort, is disproportionately devoted to assessing the benefits of prescribed medications, the evidence presented to practitioners may not be a true or valid reflection of reality.

Safe prescribing, and the prevention of adverse drug reactions, form an important part of the policy agenda [97, 98]. The importance of local and national guidelines in directing practice should not be underestimated [90, 99]. However, without further funding, prescribing guidelines will be unable to take account of the possibility that, when other risk factors are accounted for, there is both a dose-response relationship and a biologically plausible association between antibiotics prescribed and the incidence of allergic disorders in children.

## Figures and Tables

**Table 1.** Antibiotic Exposure and Risk of Eczema in Childhood

Ref.	Population	Time of Antibiotic Prescription(s)	Method	Antibiotic Use Assessed by	Number in Study	Findings/ OR	Linked to Eczema	95% CI	Comments
McKeever *et al.* 2002 [27]	Children in the English midlands, under 11 years	1^st^ year of life	Retrospective cohort.	GP records	21,129, 72.3% of available records	(aHR) 1.22	Yes	1.12-1.34	Inconsistent dose-response relationship. No data on confounding variables
Von Mutius *et al.* 1999 [40]	Dresden, children, aged 5-11	1^st^ year 1-2 courses	Survey, clinical examination, venous bloods for IgE antibodies	Retrospective questionnaires to parents	12 601	1.26	Yes	1.05-1.52	
Floistrup *et al.* 2006 [34]	Steiner school children and a comparator group, age 5-13	1^st^ year	Survey questionnaires to parents Blood samples for IgE estimation	Retrospective questionnaires to parents.	6733	1.61	Yes	1.21-2.15	
Cohet *et al.* 2005 [39]	New Zealand children age 6-7, 1584 with previous notifiable infection plus a general population group	1^st^ year	Survey. Questionnaires mailed to parents	Retrospective questionnaires to parents.	4123	1.4	Yes	1.21-1.62	Eczema not linked to infections
Kummeling *et al.* 2007 [47]	Netherlands, children up to 2 years	1^st^ 6 months, including administration to breastfeeding mothers	Prospective cohort	Retrospective questionnaires to parents.	2764	0.94	No	0.75-1.18	
Faroogqi and Hopkin 1998 [38]	Children up to 12 years in a family doctor practice in Oxfordshire, UK	1^st^ 2 years	Retrospective cohort using. GP & public health records	GP records	1934	2.04	Yes	1.53-2.72	Stronger association with broad spectrum antibiotic
Droste *et al.* 2000 [41]	Belgium, children age 7-8 years	1^st^ year	Survey questionnaires to parents. Skin prick tests	Retrospective questionnaires to parents.	1206	1.3	95% CI includes 1,uncertain	1.0-1.80	
Sariachvili *et al.* 2007 [44]	Belgium. Infants age 1 year	1^st^ year	Prospective cohort. Parents´ reports of eczema and IgE antibody measurements	Retrospective reports of mothers at 3, 6 and 12 months	976	1.1	no	0.8-1.5	
Celedon *et al.* 2002 [52]	Boston USA, at age 5 years	1^st^ year	Prospective cohort. Parents´ reports of illness	Telephone questionnaires to parents, every 2 months for 2 years	498	1.1	no	0.4-3.1	1 parent with a history of asthma or allergy. No association with allergic rhinitis
Jedrychowski *et al.* 2006 [28]	Krakow, age 1 year	Pregnancy	Prospective cohort	Questionnaires to mothers every 3 months	102	2.3	No	0.91-5.80	
McKeever *et al.* 2002 [37]	Children in the English midlands, age 11 years	Pregnancy	Retrospective cohort	Database of GP records	24 690	(aHR) 1.19	Yes	1.09-1.31	Risk increases with more courses. Similar association with hayfever
Sariachvili *et al.* 2007 [44]	Belgium. Infants age 1	Pregnancy	Prospective cohort. Parents´ reports of eczema	Retrospective reports of mothers at 5 months´ gestation & 3 months of life	976	1.8	Yes	1.2-2.7	

Papers are ordered by sample size.

**Table 2.** Antibiotic Exposure and Risk of Asthma

Ref	Population	Time of Antibiotic Prescription (s)	Method	Antibiotic Use Assessed by	Number in Study	Findings /OR	Antibiotics Associated with Asthma or Wheezing	95% CI	Comments
Marra *et al. *2006 [52]	Studies published in English	1^st^ year, at least 1 course	Meta-analysis, 4 retrospective, plus 4 prospective studies	Not specified	27 167	2.05	yes	1.41-2.99	Association stronger for retrospective studies
Ahn *et al. * 2005 [42]	Korean children, aged 7-12	1^st^ year of life	Retrospective survey	Retrospective questionnaires to parents	26 400	1.86	Yes	1.67-2.08	Similar association with wheeze in last 12 months
McKeever *et al. *2002 [27]	Children in the English midlands, age 11 years	1^st^ year of life	Retrospective cohort.	Database of GP records	21 129	(aHR) 1.99	yes	1.72-2.31	
Von Mutius *et al. *1999 [40]	Dresden, children 5-11	1^st^ year	Survey, clinical examination, venous bloods for IgE	Retrospective questionnaire to parents.	12 601	1.64	yes	1.26-2.13	
Floistrup *et al.*2006 [34]	European Steiner school children and a comparator group, age 5-13	1^st^ year	Survey of parents IgE antibody measurements	Retrospective questionnaires to parents.	6733	2.79	Yes	2.03-3.83	
Celedon *et al.*2004 [45]	Boston USA, children age 2-5, >4 courses	1^st^ year	Retrospective cohort	Record review of automated medical record systems	4408	0.9	no	0.6-1.3	
Cohet *et al. *2005 [39]	New Zealand children age 6-7, 1584 with previous notifiable infection plus a general population group	1^st^ year	Survey by mailed questionnaire	Retrospective questionnaires to parents.	4123	2.10	Yes	1.79-2.48	Asthma not linked to notifiable infections
Kummeling *et al. *2007 [47]	Netherlands. Children wheezing in 1^st^ 2 years	1^st^ 6 months, including administration to breastfeeding mothers	Prospective cohort	Retrospective questionnaires	2764	2.32	Yes	1.55-3.48	
Farooqi and Hopkin 1998 [38]	Children up to 12 in a family doctor practice in Oxfordshire, UK	1^st^ 2 years	Retrospective cohort using. GP & public health records	GP records	1934	3.19	yes	2.43-4.18	
Droste *et al. *2000 [41]	Belgium, children aged 7-8	1^st^ year	Survey questionnaires to parents. Skin prick tests	Retrospective questionnaires to parents.	1206	1.7	Confidence interval includes 1	1.0-3.1	
Illi *et al. *2001 [53]	German children aged 7 years	1^st^ 7 years	Prospective cohort. Interviews of parents, venous blood samples for IgE estimation, clinical examination	Parental report of antibiotic use	1120	1.08	no	0.59-1.99	Antibiotics for lower respiratory tract infections excluded
Cullinan *et al. *2004 [50]	Asthma in adults in Kent, UK	1^st^ 5 years	Retrospective cohort. self-report of illness as adult	GP records of prescriptions	746	1.08	yes	1.03-1.13	
Harris *et al. *2007 [54]	Children aged 8, in Kent, UK	1^st^ 5 years	Prospective cohort. Annual retrospective questionnaires to parents	GP records	523	1.07	yes	1.03-1.10	Current wheezing as end point. Association reduces when respiratory infections are discounted
Celedon *et al. *2002 [52]	Boston USA, children age 5	1^st^ year	Prospective cohort. Parents´ reports of illness	Retrospective questionnaires by telephone to parents, every 2 months for 2 years	498	0.9	no	0.4-1.8	
Wickens *et al. *1999 [51]	Children in Steiner schools in New Zealand	1^st^ year	Survey of parents	Retrospective questionnaires to parents	456	2.74	yes	1.110-6.85	
Thomas *et al. *2006 [55]	Manchester, UK, children aged 3-5	1^st^ year	Case control.	Medical records and prospective parental diaries	72	1.32	no	0.99-1.78	Association found if first 3 years´ use was considered
McKeever *et al. *2002 [37]	Children in the English midlands, under 11 years	During pregnancy >2 courses	Retrospective cohort.	Database of GP records	24 690	(aHR) 1.12	yes	1.02-1.24	Risk not significant with fewer courses.
Jedrychowski *et al. *2006 [28]	Krakow children age 1 year, non-smoking mothers	During 2^nd^ & 3^rd^ trimesters of pregnancy	Prospective cohort	Retrospective questionnaires to mothers every 3 months	102	4.42	yes	1.05-18.8	Similar association with allergic rhinitis

Papers are ordered by sample size.

**Table 3.** Antibiotic Exposure and Risk of Atopy

Ref.	Population	Time of Antibiotic Prescription (s)	Method	Antibiotic Use Assessed by:	Number in Study	Findings/ OR	Antibiotics Associated with Atopy	95% CI	Comments
Von Mutius *et al. *1999 [40]	Dresden, children aged 5-11	1^st^ year >5 courses	Survey. Questionnaires to parents, clinical examination, venous bloods for IgE	Antibiotic use assessed by retrospective questionnaires	5067	0.92	no	0.72-1.17	
Tamay *et al. *2007 [43]	Istanbul, children aged 6-12	1^st^ year	Survey	Retrospective questionnaires to parents	2387, 95% response rate	1.26	yes	1.01-1.57	Outcome was allergic rhinitis, used as marker for atopy
Floistrup *et al. *2006 [34]	Steiner school children and a comparator group, age 5-13	1^st^ year	Survey of parents. Blood samples for IgE estimation	Antibiotic use assessed by retrospective questionnaires	1856	1.15	no	0.84-1.58	
Kummeling *et al. *2007 [47]	Netherlands. Children up to 2 years	1^st^ 6 months, including administration to breastfeeding mothers	Prospective cohort	Antibiotic use assessed by retrospective questionnaires	815	1.32	No	0.86-2.02	
Cullinan *et al. *2004 [50]	Atopy (positive skin prick test) in adults in Kent	1^st^ 5 years	Retrospective cohort, self-report of illness	GP records of prescriptions	746	OR 1.01	no	0.97-1.05	No association with hayfever, but association with asthma, above
Droste *et al. *2000 [41]	Belgium, children aged 7-8	1^st^ year	Survey questionnaires to parents. Skin prick tests	Retrospective questionnaires to parents.	675	OR 1.1	no	0.7-1.7	
Harris *et al. *2007 [54]	Children aged 8, in Kent, UK	1^st^ 5 years	Prospective cohort. Annual retrospective questionnaires to parents	GP records	490	1.00	no	0.97-1.03	Atopy assessed from notes
Johnson *et al. *2005 [57]	Detroit, USA, children aged 6-7	1^st^ 6 months	Prospective cohort Clinical examination, skin prick tests, IgE from venous blood	All Medical records.	488	1.48	no	0.94-2.34	associations in subgroups

Papers are ordered by sample size. aHR, adjusted hazard ratio; OR, odds ration; CI, confidence intervals; GP, general (medical) practitioner.

## GLOSSARY

- **Antibodies. :** Immunoglobulin proteins secreted by B lymphocytes. Antibody synthesis is induced by specific antigens, and they combine with these specific antigens usually to initiate pathogenic destruction. **An antigen** is any molecule, usually a protein or polysaccharide, that interact with an antibody. The term antigen is usually used in immunology to describe foreign material although antigens can be generated within the body e.g. A and B antigens on the surface of erythrocytes.
- **Atopy.** A genetic predisposition to immediate allergic response to common environmental antigens, such as pollen, house mites. Clinical manifestations include hayfever, asthma (some types), eczema or atopic dermatitis, food and drug allergies.
- **Cytokines.** Proteins released from cells of the immune system to communicate with and regulate other cells of the immune system.
- **Histamine.** This is released by basophils, mast cells and platelets. It causes local vascular dilation, an immediate increase in vascular permeability, swelling and itching. It is released on contact with stinging nettles and in some allergies. The problems caused by histamine are ameliorated by antihistamine creams or tablets.
- **Th or helper T cells.** Lymphocytes which direct other lymphocytes by releasing cytokines. The 2 main types are Th1 and Th2.
